# Cryptococcosis in Colombia: Compilation and Analysis of Data from Laboratory-Based Surveillance

**DOI:** 10.3390/jof4010032

**Published:** 2018-03-01

**Authors:** Patricia Escandón, Jairo Lizarazo, Clara Inés Agudelo, Elizabeth Castañeda

**Affiliations:** 1 Grupo de Microbiología, Instituto Nacional de Salud, Bogotá 111321, Colombia; cia1949@gmail.com (C.I.A.); ecastaneda21@gmail.com (E.C.); 2 Departamento de Medicina Interna, Hospital Universitario Erasmo Meoz, Universidad de Pamplona, Cúcuta 540018, Colombia; jflizar@gmail.com

**Keywords:** cryptococcosis, Colombia, epidemiology

## Abstract

The passive and voluntary surveillance of cryptococcosis in Colombia since 1997 has seen an increasing participating rate, revealing its importance to both in immunosuppressed and immunocompetent people. The present work details the national data gathered in 1997–2016, through a retrospective analysis of the information collected in the survey. From a total of 1974 cases reported, an overall incidence of 0.23 cases per 100,000 people was found. This incidence rose to 1.1 cases per 1000 people in the Acquired Immunodeficiency Syndrome (AIDS) population. Cryptococcosis was most common in male young adults (26–40 years), with a male:female ratio of 3.9:1 in the general population and 5.4:1 in Human Immunodeficiency Virus (HIV) patients. Culture was the most common form of diagnosis in 96.3% of cases, recovering *C. neoformans* species in 87.5% and *C. gattii* in 3.1% of samples. VNI was the most prevalent (96.1%) molecular type, while VGII predominated in *C. gattii* isolates (54.3%). Early mortality was reported as the outcome in 47.5% of patients. Cryptococcosis remains an important opportunistic disease in Colombia and is gaining status as a primary pathogen in apparently immunocompetent patients. Our findings show the importance of including cryptococcosis as a notifiable disease, which will allow for improving opportune diagnosis and treatment, resulting in better patient outcomes.

## 1. Introduction

Cryptococcosis is being recognized as one of the most important fungal infections throughout the world, especially in immunocompromised patients, acting as an opportunistic disease. However, the etiological agents of cryptococcosis may act as primary pathogens when affecting hosts with no apparent immunosuppression. The causative agents of the disease are recognized as the *Cryptococcus neoformans* species complex, grouping the commonly known varieties (*grubii* and *neoformans*) and serotypes (A, D, and AD), and the *Cryptococcus gattii* species complex, with serotypes B and C [1]. The proposal to divide *C. neoformans* into two species and *C. gattii* into five would add knowledge on the epidemiology and genetic population of the fungus, but no consensus on this classification has been reached [1,2]. Some sporadic interspecies hybrids have been reported [3]. 

In Colombia, a laboratory-based surveillance system has been applied passively and voluntarily since 1997 and has aimed at determining demographic the characteristics of patients, risk factors associated with the disease, laboratory diagnoses, signs and symptoms, treatments, types of cryptococcosis and outcomes. The data from this system have revealed the increasing importance of this mycosis in the country to both immunocompromised and immunocompetent patients. Because cryptococcosis is not of compulsory notification in the country, data collected throughout the survey may be an underestimate of the real incidence of disease. The overall behavior of the disease in Colombian patients is very similar to that reported in the world. Information gathered from 1457 surveys in the period 1997–2010 revealed that male patients have been reported as the most affected (76%), although the male:female ratio is decreasing with the higher number of women affected by HIV, which is the main risk factor associated with cryptococcosis. The incidence rate in the general population has stayed constant over time (0.24 cases per 100,000 people), while in AIDS patients, the tendency has been to increase. *C. neoformans* var. *grubii* is constantly associated as the primary ethological agent of the disease in both immunocompetent and immunosuppressed individuals, being represented in most of the cases by the VNI molecular pattern [4,5]. Even though cryptococcosis is not under compulsory notification in Colombia, the surveillance performed in the national territory has shown that this mycosis is causing a high morbi-mortality and has been acting as a sentinel marker for HIV infection. 

In many settings, including Colombia, despite the increasing use of antiretroviral drugs, and in some cases the early diagnosis of HIV, the incidence of systemic fungal infections such as cryptococcosis continues to rise and increase the burden of the disease [6], primarily in limited settings. This is why regions such as sub-Saharan Africa reported almost 73% of cryptococcal meningitis cases in the year 2014, and more dramatically, 74% of deaths attributed to the disease were documented in this area [7]. These same authors have estimated an average worldwide cryptococcal antigenemia prevalence of 6.0% among people with a CD4 cell count below 100 cells/μL and 181,100 deaths annually in the global population caused by cryptococcal meningitis, highlighting the increasing burden of cryptococcosis associated with the HIV population. 

The aim of the present work is to describe the data obtained from the National Cryptococcosis Surveillance conducted in Colombia from 1997 through 2016 by means of a retrospective analysis of the information retrieved in the surveys. 

## 2. Materials and Methods

### 2.1. Study Design

This was a descriptive observational study in which a retrospective analysis of data gathered in the survey between 1997 and 2016 was analyzed. The survey was designed in 1997 and updated in 2012 following the guidelines established by the European Confederation of Medical Mycology with the corresponding authorization [8] (see Material S1). Each format of the survey was previously filled out by health professionals attending the patient from public and private institutions, as well as from the public health laboratories of the Colombian political divisions (departments). 

The survey is divided into eight sections, which respectively hold the following pieces of information: (1) demographic data; (2) risk factors and whether cryptococcosis was an AIDS-defining illness or not; (3) diagnosis; (4) diagnostics images; (5) clinical manifestations; (6) cryptococcosis classification documented in the survey (pulmonary, meningitis, cutaneous, bone, disseminated or infection); (7) treatment; and (8) other therapies (see Material S1).

Complete surveys and isolates or samples were sent to the study coordinating centers (Instituto Nacional de Salud in Bogotá and Corporación para Investigaciones Biológicas in Medellín), and a database was created. The data compiled over 20 years were divided into three periods for analysis purposes: 1997–2003, 2004–2010, and 2011–2016.

### 2.2. Ethical Considerations

The present study has the endorsement of the ethics committee of the Instituto Nacional de Salud, and its implementation was subjected to the principles of medical research in human beings stated in the Helsinki Declaration. Although this survey is designed to be descriptive, patient identification is totally anonymized. No additional tests were demanded beyond those required by the consulting physician. 

### 2.3. Case Definition

A case was defined when a patient had clinical findings compatible with cryptococcosis, together with one or more of the following criteria: isolation of the yeast from a normally sterile site, or from sputum, Bronchoalveolar Lavage (BAL) and skin lesions; direct visualization of blastoconidia with Indian ink in sample; and/or a capsular antigen titer ≥1:8 in sera or any titer in Cerebrospinal Fluid (CSF). Disseminated cryptococcosis is defined by (1) a positive culture from at least two different sites or (2) a positive blood culture [9]. Relapses were considered to be when a patient presented with a new clinical episode of cryptococcosis six or more months after the initial diagnosis. 

### 2.4. Epidemiological Analysis

The number of cryptococcosis cases is given for each of the geographic departments (Colombian political divisions) that submitted information, and the prevalence is given for each of those according to the population projections reported by the Departamento Administrativo Nacional de Estadística (DANE) [10]. The mean cryptococcosis incidence per year in the general population was determined likewise, using as the denominator the average national population for the study period (43,686,389). The incidence of cryptococcosis in the AIDS population was obtained using as the denominator the average number of persons living with AIDS between 1997 and 2016 (68,012) [11,12]. The frequency of each variable in the survey was determined, such as distribution by department, age groups, gender, and risk factors, among others. Cryptococcosis was clinically classified according to signs and symptoms (see Material S1). 

### 2.5. Statistical Analysis

Data were tabulated in Microsoft Excel^®^. For the numerical variables, the analysis was done with measures of central tendency. For the categorical variables, the chi-square test or Fisher’s exact test was used, with a significance of less than 0.05 and 95% confidence. A correlation between age and period of analysis was initially performed using the Shapiro–Wilk test; due to the nature of the variables, the Spearman test was applied. The analysis was performed in the data analysis and statistical software Stata 11 for Windows (Stata Corp LLC, College Station, TX, USA)^®^. 

### 2.6. Laboratory Tests and Molecular Typing

Isolates submitted to the central laboratories were confirmed using conventional techniques [13] and species differentiation was performed by culturing the isolates in canavanine–glycine–bromothymol blue agar (CGB) [13,14]. Isolates were maintained as 10% glycerol stocks at −70 °C and in sterile distilled water at room temperature. Cryptococcal antigen determination was done using the latex agglutination system with the commercial kit Meridian Bioscience CALAS^®^.

Isolates were typed to determine the molecular pattern using one or more of the following techniques: PCR fingerprinting with primers (GTG)_5_, M13 or (GACA)_4_ [14]; Restriction Fragment Length Polymorphism (RFLP) of the URA5 gene [15] and Multilocus Sequence Typing (MLST) of seven consensus genes [16]; and previous DNA extraction using the procedure described by Casali et al. [17]. Additionally, mating type was determined in a set of isolates using the specific primers described previously [18]. 

## 3. Results

**Figure 1:**
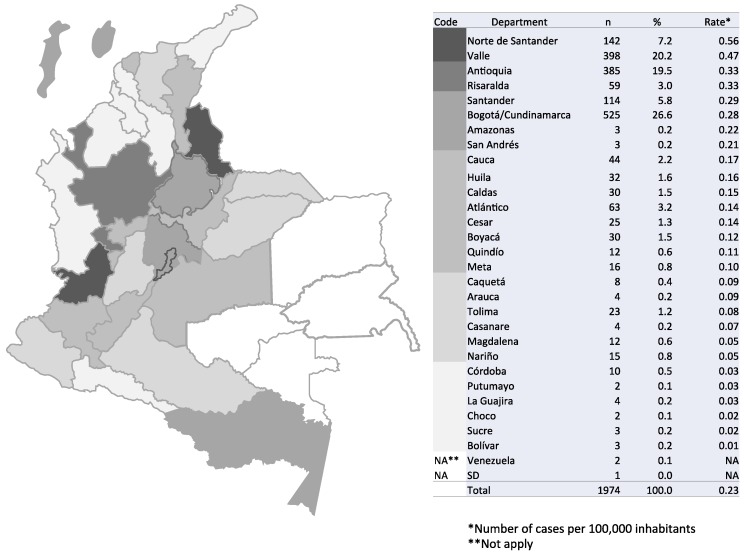
Distribution of cases of cryptococcosis in Colombia by rate and department of residence, 1997–2016.

A total of 1974 surveys were received during the 20-year study period, from 29 of the 33 political divisions (32 departments and Bogotá, the capital district, being part of the department of Cundinamarca) into which Colombia is geographically divided, as well as from two patients whose residence was in the neighboring country of Venezuela. The highest numbers of patients resided in Bogotá/Cundinamarca, Antioquia, and Valle, representing 66.3% of the grand total. No surveys were received during the 20-year period from the departments of Vichada, Guainía, Guaviare, and Vaupés. The distribution of cryptococcosis cases by department of residence and by period is shown in Figure 1 and Table S2. 

### 3.1. Incidence of Cryptococcosis in Colombia, 1997–2016

The incidence of the disease in the general population was 0.23 cases per 100,000 people. Departments with the highest incidence rate were Norte de Santander (0.56 cases per 100,000 people) and Valle (0.47 cases per 100,000 people) (Figure 1). The incidence in the period 1997–2003 was of 0.23 cases per 100,000 people, with a rise to 0.25 cases per 100,000 people for the interval 2004–2010 and a decrease to 0.17 cases per 100,000 people for the last period, 2011–2016. 

### 3.2. General Characteristics of Cryptococcosis Patients in Colombia, 1997–2016

**Figure 2:**
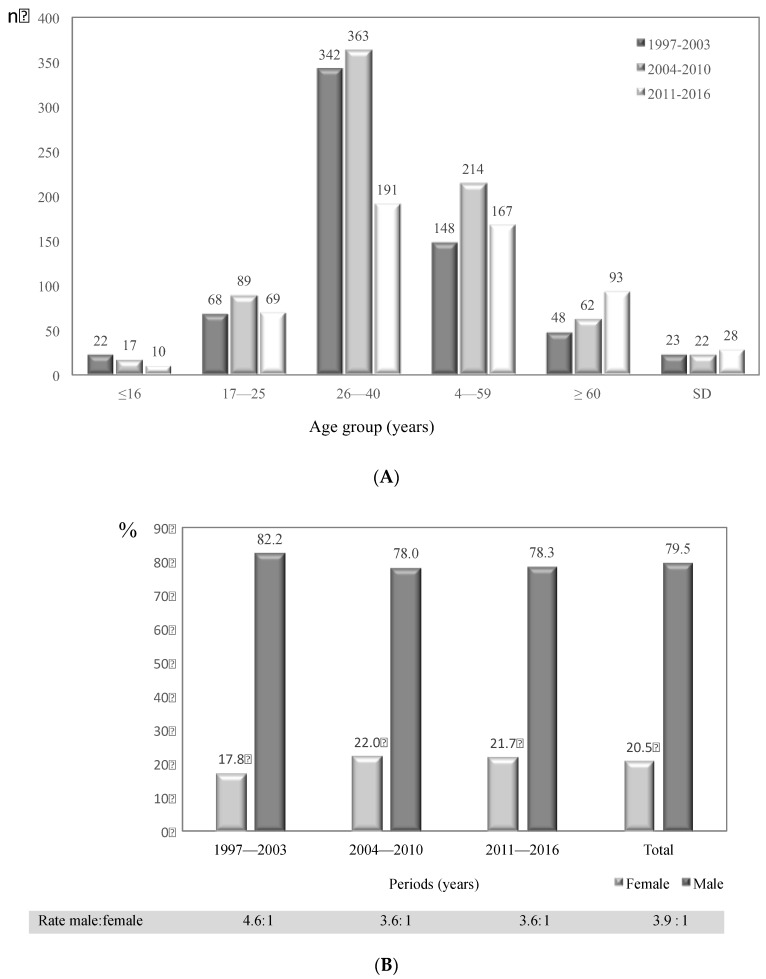
Number of patients with cryptococcosis in Colombia, 1997–2016. (**A**) Distribution by age group and study period. (**B**) Distribution by gender and study period.

The number of patients with cryptococcosis by age group and period of study analyzed is shown in Figure 2A. Of the 1974 patients, 73 had no age data. The disease was most common in the age group 26–40 years, with 877 (46.1%) cases in total. The age group 41–59 years was represented by 508 (26.7%) cases. Comparing the 26–40 age group with the other age groups revealed a statistically significant difference (*p* < 0.001). In this same age group, a significant decrease in cases was observed, from 363 in the second period to 191 in the third period (*p* < 0.001). 

For the whole period of the surveillance, males were the most affected by cryptococcosis with 79.5% of cases, a trend that remained during the three periods analyzed (Figure 2B). The male:female ratio for the 20-year period of analysis was 3.9:1. The proportions of males and females affected by cryptococcosis in each of the three periods analyzed is shown in Figure 2B, highlighting the decrease in the male:female gap from 4.6:1 during 1997–2003 to 3.6:1 during 2004–2010 (*p* < 0.001).

### 3.3. Risk Factors Associated with Cryptococcosis in Colombia, 1997–2016

Table 1 summarizes the risk factors identified in the surveys submitted during the 20-year period. The main risk factor was AIDS, which was present in 1505 (76.2%) of the total number of patients with cryptococcosis. In 248 (12.6%) cases, no apparent risk factor was identified for the occurrence of cryptococcosis. 

A total of 1505 (76.2%) surveys reported HIV/AIDS status as a risk factor for cryptococcosis. The incidence of cryptococcosis in this population for Colombia was 1.1 cases per 1000 people. 

**Figure 3:**
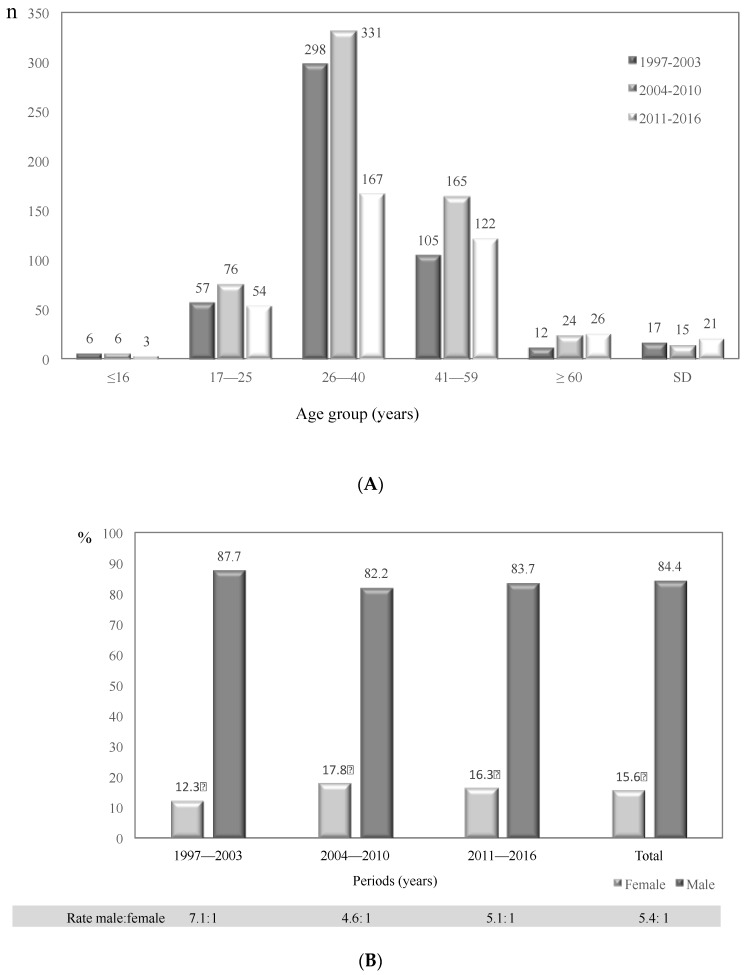
Number of HIV/AIDS patients with cryptococcosis in Colombia, 1997–2016. (**A**) Distribution by age group and study period; (**B**) Distribution by gender and study period.

Cases of cryptococcosis according to age group and study period are presented in Figure 3A. The 26–40 age group was the most affected during the three periods analyzed (Table 2). Men were the most affected by HIV/AIDS, with 1270 (84.4%) cases represented in this population, while 235 (15.6%) women were reported as HIV/AIDS positive (Figure 3B, Table 2). The male:female ratio in the HIV/AIDS population was 5.4:1. It is interesting to note that the male: female ratio decreased with the increase in the number of women living with HIV/AIDS, from 7.1:1 in the first period to 4.6:1 in the second period (Figure 3B). Cryptococcosis was an AIDS-defining illness in 361 (24.0%) of cases for the whole study period.

### 3.4. Diagnosis of Cryptococcosis Cases in Colombia, 1997–2016

Culture was reported in 1909 patients, of which 1901 (99.6%) were positive for either of the two *Cryptococcus* species. Direct examination revealed positivity in 1532/1653 (92.7%) assays. Antigenemia in CSF was reactive in 595/612 (97.2%) cases, and antigenemia serum was in 199/222 (89.6%). Table 3 describes the diagnostics tests reported in the surveys by study period. 

From the 1901 positive cultures, 1612 (84.8%) were from CSF, 192 (10.1%) from blood and 97 (5.1%) from other samples (Table S3). In some cases, *Cryptococcus* was isolated from more than one sample, e.g., CSF and blood, 108 (5.7%); CSF and BAL, 13 (0.7%), primarily. Other sources of positive cultures were skin, urine, biopsies, sputum, bone marrow, glands, and other sterile sites. 

Regarding the species involved, *C. neoformans* was recovered in 1664 (87.5%) cases, and *C. gattii* was involved in 58 cases (3.1%); in 178 (9.4%) cultures, species information was not available. Concerning the distribution of *C. neoformans* and *C. gattii* isolates recovered according to age group, it is noteworthy the fact that in patients ≤16 years there was a highly proportion of *C. gattii* (8.6%) casesmpared with the other groups. A total of 1581 isolates had serotype data, with 1525 (96.5%) serotype A, 46 (2.9%) serotype B, 4 (0.3%) serotype C, and 6 (0.4%), serotype D. 

Molecular typing of isolates was done in 334 *C. neoformans* strains, in which VNI was reported as the most prevalent pattern (*n* = 321, 96.1%), and VNII was much less common (*n* = 13, 3%). *C. gattii* was typed in 46 isolates, in which the most prevalent the molecular type was VGII (54.3%), followed by VGIII (32.6%) and VGI (13.1%), as described previously [18]. 

MLST was also applied in a set of 45 *C. neoformans* isolates, revealing that ST93 was the most prevalent (53.3%), followed in much lesser proportions by ST5, ST69, ST71, ST2, ST40, ST6, ST63 and ST77. MLST was performed in 45 *C. gattii* isolates, identifying 16 different STs, with ST25 being the most prevalent (37.7%). 

Mating type was determined in 130 isolates. Mating type α was the most prevalent (83.1%) and mating type a was much less prevalent (16.9%). 

### 3.5. Diagnostic Images

Chest X-rays were performed in 858 (43.5%) cases, with normal results in 390 (45.5%) and abnormalities (infiltrations, cavitations, calcifications, nodular lesions and opacity) in 381 (44.4%). In 87 (10.1%) patients, no results were submitted. Cerebral magnetic resonance imaging and cranial computed tomography were reported in 511 (25.9%) patients, with normality in 121 (23.7%) cases, and abnormalities (brain mass, hydrocephaly, stroke, cerebral atrophy, edema, dilated Virchow-Robin spaces and cryptococcomas) in 342 (66.9%). In 48 (9.4%) surveys, no results were reported. 

### 3.6. Clinical Manifestations

Table S4 describes the clinical features of Colombian cryptococcosis patients by age group over the 20-year period of study. Headache (73.3%), fever (53.3%), nauseas and vomiting (48.6%) and confusion (40.3%) were the most prevalent symptoms in the total population. Neurological manifestations (neck stiffness, communicating and obstructive hydrocephaly, intracranial hypertension and seizures) were reported in 911 (46.1%) cases. Neurological signs were reported in 710/1505 (47.2%) of AIDS patients with cryptococcosis. According to the associated *Cryptococcus* species complex, neurological manifestations were present in 743/1664 (44.6%) of the total cases caused by *C. neoformans*, while in those caused by *C. gattii,* meningeal signs were observed in 35/58 (62.1%) cases. 

### 3.7. Cryptococcosis Classification

Classification of disease according to clinical manifestations is reported in Table 4. The most common form of clinical presentation of the disease among the 1974 patients was neurocryptococcosis in 1600 (81.1%), both in AIDS and in non-AIDS patients, followed by disseminated cryptococcosis. 

### 3.8. Antifungal Treatment and Other Therapies

Treatment options were reported in 1480 (75.0%) cases; 1243 (84.0%) patients received amphotericin B only (*n* = 782, 62.9%) or received amphotericin B in combination with fluconazole (*n* = 414, 33.3%), 5-flucytosine (*n* = 29, 2.4%), itraconazole (*n* = 15, 1.3%), or other antifungals (*n* = 3, 0.3%). Fluconazole was prescribed in 187 (12.6%) cases as the only antifungal treatment for cryptococcosis and in combination with 5-flucytosine, itraconazole or nystatin in eight (0.6%) patients. Other antifungal drugs, such as 5-flucytosine and itraconazole were reported in 42 (2.8%) cases as the only antifungal used. 

Regarding other therapies provided, antiretroviral drugs were reported in 212 (10.7%) cases; anti-tuberculosis agents in 64 (3.2%) and steroids in 34 (1.7%).

### 3.9. Mortality and Relapses

In 610 surveys, patient outcome was reported. In 290 patients (47.5%), early mortality was the outcome. Of all age groups, the 26–40-year-old group had the highest mortality at 44.3% (124 cases out of 280) of the reported outcomes in this group. AIDS patients were the population with the highest reported mortality rate, at 48.1% (227 of 472 patients). *C. neoformans* was the most prevalent causative agent in patients who were reported dead, at 85.9% (249 of 290 patients). A total of 204 (70.3%) of the patients who died were receiving treatment, primarily amphotericin B in 118 (57.8%) cases. 

Relapses were reported in 40 (2.0%) patient surveys, with 37 (90.0%) associated with HIV positive patients. Of these patients, seven (17.5%) died. 

## 4. Discussion

The passive surveillance that has been conducted in Colombia since 1997 has revealed the increasing importance of cryptococcosis, not only in the immunosuppressed population but also as an important primary pathogen in apparently immunocompetent patients. This retrospective analysis describes the incidence of the disease throughout Colombia, highlighting some departments as those that contributed with the highest numbers of patients affected by cryptococcosis, such as the capital district of Colombia, Antioquia, Norte de Santander, and Valle. The numbers are biased by the fact that in Colombia, such as most parts of the world, cryptococcosis is not under compulsory notification, and the surveillance is totally passive and voluntary. Despite the disease sub-reports and the fact that many surveys are not completely filled out, the overall incidence of cryptococcosis in Colombia during the 20-year period analyzed suggests that although there have been advances in the access of patients to highly antiretroviral therapy and early testing for HIV, the disease is still being diagnosed in a considerable number of patients. Norte de Santander has steadily had the highest incidence rate of cryptococcosis in Colombia. This highest incidence is attributed primarily to the active surveillance conducted in a third-level hospital located in the capital city of the department [15,19,20,21].

Cryptococcosis was reported in all ages, from newborns to elderly. We found 49 patients younger than 16 years, with most of them being diagnosed between 1997 and 2010, as previously reported [22]. This low incidence is consistent with the overall reports that cryptococcosis is an unusual event in children [23,24]. Young adults were the most affected by the disease, with a significant difference compared with the other age groups, consistent with the fact that young adults are most affected by HIV. 

As occurs worldwide, in Colombia cryptococcosis is reported more frequently in men than in women, independent of the tendency for men to be the most affected by HIV. However, this viral immunosuppression may explain the decrease in male:female ratio of cryptococcosis from the period 1997–2003 period to the 2004–2010 period among AIDS patients [25,26]. Historically, as in most parts of the world, the main risk factor associated with cryptococcosis in Colombian patients is HIV seropositivity. The fact that over the 20-year period of analysis, 76.2% of our patients with cryptococcosis were also affected by AIDS resembles the situation reported not only in sub-Saharan Africa, where the major burden of meningocryptococcosis cases occur in the HIV population but also in regions of North America [27] and South America [7,28,29,30], among others. This finding is contrary to that reported in Australia and New Zealand by the Australasian Cryptococcal Study Group, where the incidence of AIDS-associated cases declined every year because cryptococcosis is an important disease in the aboriginal immunocompetent population [31]. As mentioned by O’Halloran et al., the group of HIV-negative patients diagnosed with cryptococcal disease is very heterogeneous, with different degrees of immunosuppression; in Colombia, we see patients with risk factors in much lower proportions than those in HIV patients who were under corticosteroids therapy, transplanted patients or patients with diabetes, among others, and a substantial percentage (12.6%) of individuals with an apparently normal immune system [32].

The ease in diagnosing the disease by morphological and physiological tests allowed us to recover the fungus in almost 97.0% of the cases, and as expected, the majority of the isolates were associated with CSF, which is considered the sample of choice for the diagnosis of cryptococcosis. CSF sampling must be done by lumbar puncture with measurement of the opening pressure in those patients with a suspicion of cryptococcal meningitis [33,34]. On the other hand, testing the CSF cryptococcal circulating antigen is very sensitive and specific for the diagnosis of meningitis and a very useful tool in those patients with a negative India ink test, providing a suggestion of the presence of the disease well the before the yeast grows in culture. A reactivity of antigenemia in CSF in approximately 97.0% of our patients agrees with this previous finding and highlights the importance of using the three-gold standard diagnostic tests for an accurate diagnosis of cryptococcosis. In its guidelines for the diagnosis, the WHO has included prevention and management of cryptococcal disease the cryptococcal antigen (CrAg) screening in severely immunocompromised patients (CD4 count <100 cells/mm^3^) as a strategy to prevent or detect at an early stage a significant number of meningitis cases, together with a preventive antifungal treatment [35]. Though this strategy has been proven effective in over 20 countries around the world, being highly cost-effective and successful at reducing mortality [36,37], very few countries have committed resources and actions to implementing this program routinely in patients. Colombia has made some attempts at showing the usefulness of early detection of cryptococcosis in patients with a depleted CD4 count. The first report, long before the point-of-care tests were launched, was done in 2002 by Lizarazo et al., who suggested that patients with CD4 count <100 cells/mm^3^ are at risk of developing the disease, in which the presence of this mycotic infection could be established even in the absence of symptoms [38]. More recently, two retrospective analyses have been done with Colombian samples, in which the usefulness of the commercially rapid test available for the screening of the disease was evidenced over the common detection of the cryptococcal antigen by latex agglutination [39,40]. Despite these reports, the usefulness of this test has not been recognized by the public health authorities in Colombia and is only sporadically requested by physicians.

Among all the cultures received, the most frequent species recovered was *C. neoformans*, predominantly represented by var. *grubii* molecular type VNI, as reported in other parts of the world, where this specific variety and genotype is responsible for the vast majority of cases, in both immunosuppressed and immunocompetent patients, except in Oceania, were the epidemiology of the disease is predominated by *C. gattii* [7,41]. Serotype D of *C. neoformans* var. *neoformans* was found very rarely (0.4%) and is considered an uncommon isolate in our region [41]. The few isolates recovered from clinical sources in Colombia were recently included in a global genetic population structure that included isolates from different origins and sources, showing that this is a highly recombinant population characterized by high variability [42]. 

As reported previously by our group, cryptococcosis cases caused by *C. gattii* are less common in Colombian patients and are found in only 3.1% of cases, most of which are associated with the molecular type VGII [19]. Patients less than 16 years of age were mostly affected by *C. gattii,* an interesting finding that needs to be further studied, keeping in mind that cryptococcosis in children, especially caused by *C. gattii,* is considered an unusual and unexplainable event [22,23,24].

Diagnostic images are an important tool in the correct identification of the disease. Radiographic imaging of the brain using either computed tomography or magnetic resonance should be performed in those patients presenting with focal neurological signs or symptoms, such as AIDS patients. However, cerebral imaging in our patients was only reported in approximately 26.0%, with abnormalities in more than half of them, suggesting that this routine examination is an important complement when diagnosing the disease [43]. 

Clinical manifestations in patients affected by cryptococcosis are similar, notwithstanding the degree of immunosuppression in the individual. As commonly reported, Colombian patients manifest their disease mostly with headache, fever, nausea and vomiting and confusion, with no preference according to age, a finding that is directly related to the fact that the majority of cases had neurocryptococcosis as the most common form of clinical presentation. Clinical manifestations in HIV and non-HIV patients have been documented [44]. In general, more HIV-uninfected patients present with visual symptoms and are more likely to present altered mental status and seizures when compared to HIV-infected patients, which is also consistent with infection caused by *C. gattii*, as seen in our study in which mental changes and visual alterations were important clinical findings [19]. It is believed that episodes of cryptococcosis due to *C. gattii* tend to be more virulent than those caused by *C. neoformans*, having the capacity to cause multiple granulomas in the brain and lungs and evade the adaptive immunity of the host [45,46,47]. We suggest that patients with cryptococcosis caused by *C. gattii* present more severe clinical manifestations due to a late diagnosis; most of these patients are not HIV carriers and therefore clinicians delay the diagnosis [48]. However, the data collected in this Colombian survey do not allow for such a conclusion, since timing in symptoms presentation is not inquired. 

WHO guidelines recommend that treatment for cryptococcosis should cover three phases: induction (amphotericin B combined with fluconazole or flucytosine), a consolidation phase (fluconazole for eight weeks), and, finally, a maintenance or suppressive treatment phase (fluconazole) [35]. In Colombia, this regimen is not met in most of our patients because of costs issues, since as reported in the 1480 surveys where treatment data were available, approximately 63.0% of patients received amphotericin B as the only antifungal treatment, 33.3% received it combined with fluconazole, and barely 2.4% received flucytosine as an induction treatment phase. Emphasis on providing appropriate and timely treatment to cryptococcal patients, together with an approach leading to early diagnosis of the disease is needed to reduce mortality rates, especially in AIDS patients. Currently, new simplified treatment regimens with short combinations of amphotericin B and oral combination of fluconazole and flucytosine are being tested in African-low and middle-income countries, which could be cost-effective and, more importantly, provide an improved and simplified treatment for cryptococcal meningitis that could be more accessible to reduced limited populations where disease burden is highest [33]. Most patients were infected with HIV, had advanced immunodeficiency on admission, and were not receiving antiretroviral therapy or had poor adherence to treatment. In addition, many patients presented severe forms of cryptococcosis, including disseminated forms, which could explain the high mortality despite early diagnosis and antifungal therapy.

As mentioned recently by Molloi et al., although HIV/AIDS is certainly not neglected, opportunistic infections such as cryptococcosis are far from a priority in public health systems despite meeting the criteria of the World Health Organization for a Neglected Tropical Disease (affects the poor disproportionately, causes high rates of mortality and morbidity, primarily affects persons living in the tropics and sub-tropics, its control or eradication is a priority, and it is practically ignored in research areas [33]). However, local and regional efforts need to be made in order to press authorities for funding and policies targeted to the implementation of CrAg-screening programs and access to adequate antifungal treatments such as 5-flucytosine and liposomal amphotericin B, all efforts that aim to provide our patients with an accurate diagnosis of cryptococcosis and better treatment strategies that will improve patient outcomes.

## Figures and Tables

**Table 1:** Risk factors associated with cryptococcosis cases in Colombia, 1997–2016. Comparison between study periods.

Risk Factor	1997–2003	2004–2010	2011–2016	Total	
	*n*			*n*	%
AIDS	495	617	393	1505	76.2
Corticosteroids	19	32	22	73	3.7
Autoimmune disease	2	10	8	20	1.0
Transplantation	8	2	8	18	0.9
Tumor	12	7	7	26	1.3
Diabetes	3	4	11	18	0.9
Cirrhosis	1	2	1	4	0.2
Chronic renal failure	2	2	6	10	0.5
Unknown or No risk factor	88	80	80	248	12.6
Others	21	9	22	52	2.6
Total	651	765	558	1974	100.0

**Table 2:** Distribution of AIDS patients with cryptococcosis in Colombia, 1997–2016, according to age, sex, and study period.

Age Group	1997–2003				2004–2010				2011–2016				Total				Total *
	Male		Female		Male		Female		Male		Female		Male		Female		
	*n*	%	*n*	%	*n*	%	*n*	%	*n*	%	*n*	%	*n*	%	*n*	%	*n*
≤16	3	50.0	3	50.0	4	66.7	2	33.3	3	100.0	0	0.0	10	66.7	5	33.3	15
17–25	44	77.2	13	22.8	59	77.6	17	22.4	45	83.3	9	16.7	148	79.1	39	20.9	187
26–40	262	91.0	36	9.0	276	83.4	55	16.6	141	84.4	26	15.6	679	85.3	117	14.7	796
41–59	98	93.3	7	6.7	139	84.2	26	15.8	102	83.6	20	16.4	339	86.5	53	13.5	392
≥60	12	100.0	0	0.0	19	79.2	5	20.8	23	88.5	3	11.5	54	87.1	8	12.9	62
SD	15	88.2	2	11.8	10	66.7	5	33.3	15	71.4	6	28.6	40	75.5	13	24.5	53
Total	434	87.7	61	12.3	507	82.2	110	17.8	329	83.7	64	16.3	1270	84.4	235	15.6	1505

* Total patients with AIDS data.

**Table 3:** Diagnostic procedures used in Colombian cryptococcosis patients, 1997–2016.

Diagnostic Method	Study Period			Total	
	1997–2003	2004–2010	2011–2016		
	*n*			*n*	%
Culture	612	738	551	1901	96.3
+ direct examination	359	397	202	958	48.5
+ antigenemia + direct examination	180	231	130	541	27.4
only culture	55	75	175	305	15.5
+ antigenemia	18	35	44	97	4.9
Antigenemia	32	17	6	55	2.8
only antigenemia	25	10	5	40	2.0
+ direct examination	7	7	1	15	0.8
Direct examination	7	10	1	18	0.9
Total	651	765	558	1974	100.0

+ (plus) Refers to the use of an additional diagnostic test besides culture or antigenemia.

**Table 4:** Clinical presentation of cryptococcosis cases in Colombia, 1997–216.

Clinical Presentation	AIDS Condition						Total	
	Positive		Negative		SD			
	*n*	%	*n*	%	*n*	%	*n*	%
Neurocryptococcosis/meningitis	1218	80.9	319	82.2	64	79.0	1601	81.0
Disseminated	206	13.7	42	10.8	14	17.3	262	13.3
Pulmonary	46	3.1	14	3.6	2	2.5	62	3.1
Cutaneous	6	0.4	1	0.3	1	1.2	8	0.4
Ganglion	3	0.2	0	0.0	0	0.0	3	0.2
Oropharyngeal	2	0.1	0	0.0	0	0.0	2	0.1
Peritoneal	2	0.1	0	0.0	0	0.0	2	0.1
No classified	22	1.5	12	3.1	0	0.0	34	1.7
Total	1505	100.0	388	100.0	81	100.0	1974	100.0

## Supplementary Materials

The following are available online at http://www.mdpi.com/2309-608X/4/1/32/s1. Material S1: Epidemiological survey on cryptococcosis in Colombia; Table S2: Distribution of cryptococcosis cases by residence department and by period analyzed; Table S3: Distribution of samples processed and positive for culture by period in patients affected by cryptococcosis in Colombia, 1997–2016; Table S4: Distribution of symptoms by age group in patients affected by cryptococcosis in Colombia, 1997–2016.
