# Supplementary material for: Cryptococcosis in Colombia: Compilation and Analysis of Data from Laboratory-Based Surveillance
Source: J Fungi (Basel). 2018 Mar 1;4(1):32. doi: 10.3390/jof4010032 (PMC5872335; doi:10.3390/jof4010032)
Supplement: Supplementary file 1 [file jof-04-00032-s001.zip › Material S1.pdf]

INS-CIB

El paciente provee consentimiento informado verbal para el diligenciamiento de esta encuesta SI____ NO_____

ENCUESTA EPIDEMIOLÓGICA SOBRE LA CRIPTOCOCOSIS EN COLOMBIA

Datos del paciente

Nombre y apellido (iniciales) ____________ Sexo M ____ F ____

Año de nacimiento ____ ____ ____ ____ Nº documento de identidad ____________

Departamento de nacimiento ____________ Departamento de residencia ____________ Teléfono ____________

**Factores de riesgo** *(marque con una X la opción que corresponda)* *(cuando la respuesta es SI, por favor complete la información)*		**Si**	**No**	**No dato**
1. Infección por VIH				
Número de células CD4+/mL				
2. Uso de corticosteroides				
3. Enfermedad autoinmune				
¿Cuál?				
4. Trasplante				
Órgano trasplantado				
5. Tumor sólido				
Tipo de tumor				
6. Malignidad hematológica				
Tipo de malignidad				
7. Diabetes mellitus				
8. Cirrosis hepática				
9. Falla renal crónica				
10. Sarcoidosis				
11. Otro factor				
¿Cuál?				
12. Sin factor de riesgo aparente				
¿Fue la criptococosis la enfermedad que definió el sida?				

Diagnóstico de criptococosis

Fecha Día ____ Mes ____ Año ____ ☐ Caso nuevo ☐ Recaída

**Prueba diagnóstica** *(si se realizó la prueba, marque con una X el resultado, si no, marque no realizada)* *(cuando la respuesta es SI, por favor complete la información)*		**Positiva**	**Negativa**	**No realizada**
1. Tinta china en el LCR				
2. Antígeno capsular en el LCR				
Título				
3. Antígeno capsular en suero				
Título				
4. Cultivo para *Cryptococcus neoformans*				
**¿De qué muestra?**	LCR			
	Sangre			
	Lavado broncoalveolar			
	Orina			
	Piel			
	Otro (especificar)			
5. Estudio histopatológico				
¿De cuál órgano o tejido?				

**Imágenes diagnósticas** *(marque con una X la opción que corresponda)*		**Normal**	**Anormal**	**No dato**
Radiografía del tórax				
**¿Resultado anormal?**	Infiltrados alveolares			
	Cavitaciones			
	Derrame pleural			
	Calcificaciones			
	Masa pulmonar			
	Otra (especificar)			

Imágenes cerebrales diagnósticas (*marque con una X la opción que corresponda*)	Si	No	No dato
1. TAC de cráneo			
2. Resonancia magnética (RM) de cerebro			

Resultados de los estudios de imágenes cerebrales (*marque con una X la opción que corresponda*)

Anormalidad	TAC	RM		Anormalidad	TAC	RM
1. Masa cerebral				4. Infarto		
2. Cerebritis				5. Atrofia cerebral		
3. Hidrocefalia				6. Dilatación de los espacios de Virchow-Robin		
Otra (especificar):						

Manifestaciones clínicas (*marque con una X la opción que corresponda*)	Si	No	No dato
1. Cefalea			
2. Náuseas o vómito			
3. Fiebre (temperatura ≥ 38°C)			
4. Confusión o cambios mentales			
5. Tos (de 3 ó más días de evolución, con o sin expectoración)			
6. Pérdida de visión			
7. Visión doble			
**8. Signos neurológicos focales (especificar):**<br>a. Rigidez nucal y signos meníngeos (Kernig y Brudzinski)			
b. Hidrocefalia comunicante			
c. Hidrocefalia obstructiva			
d. Hipertensión intra-craneana sin hidrocefalia<br>Valor de la presión de apertura del LCR en mm:			
e. Convulsiones			
f. Pérdida de peso			
g. Otras (especificar)			

Clasificación de la criptococosis (*marque con una X la opción que corresponda*)

B45.0 - J99.8 Criptococosis pulmonar		B45.7 Criptococosis diseminada o generalizada	
B45.1 - G05.2-G02.1. Criptococosis cerebral, meningitis, meningocerebral		B45.8 Criptococosis especificada NCOP	
B45.2 - L99.8. Criptococosis cutánea o de la piel		B45.9 Criptococosis (infección)	
B45.3 - M90.2. Criptococosis de hueso u ósea			

Tratamiento para la criptococosis

	Medicamentos			Dosis/ día			Tiempo		
Inicial	1.	2.	3.	1.	2.	3.	1.	2.	3.
Mantenimiento	1.	2.	3.	1.	2.	3.	1.	2.	3.
Profilaxis 2°	1.	2.	3.	1.	2.	3.	1.	2.	3.

Resultado de la terapia (egreso) (*marque con una X la opción que corresponda*)

☐ Éxito (vivo)

☐ Falla ☐ Recaída ☐ Tiempo de recaída

☐ Muerte ☐ Tiempo de muerte (*tiempo de muerte luego del diagnóstico*).

☐ **No dato**

Otras terapias

☐ Anti-TB ☐ Antiretroviral ☐ Esteroides ☐ **Otra (¿cuál?)**

Profesional responsable de la información

Nombre ______ Dirección ______

Teléfono ______ FAX ______ E-mail ______

Fecha de envío ______

Nota: favor enviar este formulario por correo electrónico a: Patricia Escandón pescandon@ins.gov.co y p_escand@yahoo.com y el aislamiento (especificar de donde se recuperó) al laboratorio de Micología del Instituto Nacional de Salud, Avenida calle 26 #51-60, Bogotá, Colombia. Teléfono (1) 220 77 00 Ext 1421. La información recolectada en esta encuesta tendrá un manejo de forma anónima y confidencial.
