# Supplementary material for: Cryptococcosis in Colombia: Compilation and Analysis of Data from Laboratory-Based Surveillance
Source: J Fungi (Basel). 2018 Mar 1;4(1):32. doi: 10.3390/jof4010032 (PMC5872335; doi:10.3390/jof4010032)
Supplement: Supplementary file 1 [file jof-04-00032-s001.zip › S5. Colombian Cryptococcosis Study Group.pdf]

Colombian Cryptococcosis Study Group
Antioquia:
Catalina deBedout, Angela Restrepo, Corporación para Investigaciones Biológicas
Angela Tobón, Carlos Agudelo, Carlos Restrepo, Hospital La María
Myrtha Arango, Universidad de Antioquia
Dora Rivas, Luz Marina Melquizoz, Victoria García, Paula Agudelo, Hospital General de Medellín
Ana María Restrepo, Clínica CES
Maryan Vásquez, Magda Cárdenas, Mayiber Henao, Edna Vásquez, Clínica Saludcoop Medellín
Carlos Agudelo, Alejandro Vélez, Carlos Ignacio Gómez, Sergio Jaramillo, Hospital Pablo Tobón Uribe

Atlántico: María Clara Noguera, Universidad Metropolitana
Adriana Marín, Clínica General del Norte
Samir Viloria, Hospital Universidad del Norte.
Bogotá:
María Isabel Medina, Hospital Simón Bolívar
Carlos Álvarez, Judy Andrea Puerta, Claudia Linares, Maritza Rojas, Hospital San Ignacio
Gloria Inés Gallo, Hospital Santa Clara
Claudia Clavijo, Clínica San Rafael
Martha Isabel Garzón, Karen Melissa Ordoñez, Hospital El Tunal
Claudia Clavijo, Clínica San Rafael
Sandra Nuñez, Gloria Inés Gallo, LSP Bogotá
Luz Mery Jiménez, Hospital La Victoria
Andrea López Guachetá, Hospital Meissen
Nubia Escobar, Claudia Pardo, Hospital de Kennedy
Rossana Mejía, Clínica Nueva
Caldas:
Martha Cecilia Kogson, Gilberto Manjarres, Hospital Santa Sofía
Cauca: Fabiola González, Unversidad del Cauca
Cesar: José Yesid Rodríguez, Soraya Morales
Huila:
Luis Fernando Duran, Hospital Universitario de Neiva
Meta:
Sandra Hurtado, Hospital Departamental de Villavicencio
Norte de Santander:
Yeni Peña, Hospital Universitario Erasmo Meoz
Risaralda:
Myriam Gómez, Berenice Isaza, Hospital San Jorge
Valle:
Claudia Rocío Castañeda, Mónica Recalde, Juan Diego Vélez, Juan Carlos Alvir, Álvaro Iván Muñoz, Fernando Rozo, Fundación Valle del Lilí
Nancy Villamarín, Johan Castrillón, María Inés Alvárez, Hospital Universitario del Valle
