# Supplementary material for: Cryptococcosis in Colombia: Compilation and Analysis of Data from Laboratory-Based Surveillance
Source: J Fungi (Basel). 2018 Mar 1;4(1):32. doi: 10.3390/jof4010032 (PMC5872335; doi:10.3390/jof4010032)
Supplement: Supplementary file 1 [file jof-04-00032-s001.zip › Table S2 .docx]

Supplementary table 2. Distribution of cryptococcosis cases by residence department and by period analyzed.

| Department | Periods | | | Total | |
| --- | --- | --- | --- | --- | --- |
|  | 1997-2003 | 2004-2010 | 2011-2016 |  |  |
|  | n | | | n | % |
| Valle | 122 | 164 | 112 | 398 | 20.2 |
| Antioquia | 120 | 204 | 61 | 385 | 19.5 |
| Bogotá/Cundinamarca | 237 | 167 | 121 | 525 | 26.6 |
| Norte de Santander | 44 | 63 | 35 | 142 | 7.2 |
| Santander | 16 | 36 | 62 | 114 | 5.8 |
| Atlántico | 7 | 14 | 42 | 63 | 3.2 |
| Risaralda | 35 | 12 | 12 | 59 | 3.0 |
| Cauca | 10 | 6 | 28 | 44 | 2.2 |
| Huila | 13 | 16 | 3 | 32 | 1.6 |
| Boyacá | 7 | 9 | 14 | 30 | 1.5 |
| Caldas | 7 | 22 | 1 | 30 | 1.5 |
| Cesar | 0 | 4 | 21 | 25 | 1.3 |
| Tolima | 8 | 10 | 5 | 23 | 1.2 |
| Meta | 1 | 9 | 6 | 16 | 0.8 |
| Nariño | 3 | 6 | 6 | 15 | 0.8 |
| Magdalena |  | 5 | 7 | 12 | 0.6 |
| Quindío | 2 | 3 | 7 | 12 | 0.6 |
| Córdoba | 6 | 4 | 0 | 10 | 0.5 |
| Caquetá | 3 | 4 | 1 | 8 | 0.4 |
| La Guajira | 0 | 0 | 4 | 4 | 0.2 |
| Arauca | 2 | 2 | 0 | 4 | 0.2 |
| Casanare | 1 | 1 | 2 | 4 | 0.2 |
| Bolívar | 2 | 0 | 1 | 3 | 0.2 |
| Sucre | 0 | 1 | 2 | 3 | 0.2 |
| San Andrés | 1 | 1 | 1 | 3 | 0.2 |
| Amazonas | 1 | 1 | 1 | 3 | 0.2 |
| Choco | 1 | 0 | 1 | 2 | 0.1 |
| Putumayo |  | 1 | 1 | 2 | 0.1 |
| Venezuela | 1 | 0 | 1 | 2 | 0.1 |
| SD | 1 | 0 | 0 | 1 | 0.0 |
| Total | **651** | **765** | **558** | **1974** | 100.0 |
