# Supplementary material for: Cryptococcosis in Colombia: Compilation and Analysis of Data from Laboratory-Based Surveillance
Source: J Fungi (Basel). 2018 Mar 1;4(1):32. doi: 10.3390/jof4010032 (PMC5872335; doi:10.3390/jof4010032)
Supplement: Supplementary file 1 [file jof-04-00032-s001.zip › Table S3.docx]

Table supplementary 3. Distribution of samples processed and positive for culture, by period in patients affected by cryptococcosis in Colombia, 1997-2016

| Type of sample | Periods | | | Total |
| --- | --- | --- | --- | --- |
|  | **1997-2003** | **2004-2010** | **2011-2016** |  |
| CSF* | 521 | 588 | 381 | **1490** |
| CSF+Blood | 30 | 30 | 42 | **102** |
| CSF+Blood+BAL | 1 | 2 | 1 | **4** |
| CSF+Blood+skin | 1 | 0 | 1 | **2** |
| CSF+BAL | 1 | 6 | 4 | **11** |
| CSF+BAL+urine | 0 | 1 | 0 | **1** |
| CSF+BAL+skin | 1 | 0 | 0 | **1** |
| CSF+skin | 1 | 0 | 0 | **1** |
| Blood | 26 | 62 | 93 | **181** |
| Blood+BAL | 0 | 5 | 3 | **8** |
| Blood+BAL+urine | 0 | 0 | 1 | **1** |
| Blood+skin | 0 | 1 | 0 | **1** |
| Blood+ myeloculture | 0 | 0 | 1 | **1** |
| BAL | 10 | 23 | 10 | **43** |
| BAL+skin | 0 | 1 | 0 | **1** |
| Biopsy lung | 5 | 4 | 4 | **13** |
| Skin | 4 | 5 | 2 | **11** |
| Biopsy other samples | 6 | 1 | 0 | **7** |
| Other sterile body fluids | 1 | 1 | 3 | **5** |
| Urine | 1 | 1 | 2 | **4** |
| Lymph nodes |  | 3 | 1 | **4** |
| Sputum | 2 | 1 |  | **3** |
| Bone marrow |  | 1 | 1 | **2** |
| Soft palate | 1 |  |  | **1** |
| Pharynx |  | 1 |  | **1** |
| Intra-abdominal collection |  | 1 |  | **1** |
| Tracheal aspirate |  |  | 1 | **1** |
| Total | 612 | 738 | 551 | **1901** |

CSF: cerebrospinal fluid

BAL: Broncho Alveolar Lavage

Note: 8 CSF cultures were negative, 6 from the first period, 1 from the second and one from the third
