# Supplementary material for: Cryptococcosis in Colombia: Compilation and Analysis of Data from Laboratory-Based Surveillance
Source: J Fungi (Basel). 2018 Mar 1;4(1):32. doi: 10.3390/jof4010032 (PMC5872335; doi:10.3390/jof4010032)
Supplement: Supplementary file 1 [file jof-04-00032-s001.zip › Table S4.docx]

Table supplementary 4. Distribution of symptoms by age group in patients affected by cryptococcosis in Colombia, 1997-2016

| Symptoms | Age groups in years | | | | | | Total  n=1974 | |
| --- | --- | --- | --- | --- | --- | --- | --- | --- |
|  | **≤16**  n=49 | **17-25**  n=226 | **26-40**  n=894 | **41-59**  n=529 | **≥ 60**  n=203 | **SD**  n=73 |  |  |
|  | n | | | | | | n | % |
| Headache | 29 | 183 | 692 | 396 | 100 | 46 | 1446 | 73.3 |
| Fever | 24 | 130 | 497 | 286 | 82 | 33 | 1052 | 53.3 |
| Nausea and vomiting | 25 | 131 | 467 | 244 | 56 | 36 | 959 | 48.6 |
| Confusion | 19 | 74 | 346 | 236 | 94 | 26 | 795 | 40.3 |
| Meningeal signs | 14 | 66 | 202 | 127 | 28 | 13 | 450 | 22.8 |
| Cough | 6 | 51 | 214 | 107 | 45 | 10 | 433 | 21.9 |
| Abnormal vision | 10 | 49 | 164 | 87 | 16 | 10 | 336 | 17.0 |
| Seizures | 13 | 46 | 151 | 76 | 20 | 9 | 315 | 16.0 |
| Weight loss | 4 | 25 | 106 | 71 | 25 | 10 | 241 | 12.2 |
| Hydrocephalus | 7 | 18 | 102 | 51 | 20 | 6 | 204 | 10.3 |
| Neurological focalization | 7 | 20 | 84 | 41 | 19 | 3 | 174 | 8.8 |
| Neck stiffness | 3 | 19 | 60 | 48 | 15 | 8 | 153 | 7.8 |
| Cranial hypertension | 0 | 20 | 37 | 31 | 10 | 6 | 104 | 5.3 |
| Skin lesions | 0 | 8 | 18 | 16 | 3 | 0 | 45 | 2.3 |
| Growth of lymph nodes | 0 | 6 | 16 | 8 | 4 | 0 | 34 | 1.7 |
| Communicating hydrocephalus | 0 | 0 | 6 | 8 | 3 | 0 | 17 | 0.9 |
| Visual alterations | 1 | 1 | 4 | 3 | 1 | 1 | 11 | 0.6 |
| Obstructive hydrocephalus | 0 | 1 | 3 | 3 | 2 | 0 | 9 | 0.5 |
| Other meningeal signs | 0 | 1 | 2 | 6 | 0 | 0 | 9 | 0.5 |
